# Orexin A Affects INS-1 Rat Insulinoma Cell Proliferation via Orexin Receptor 1 and the AKT Signaling Pathway

**DOI:** 10.1155/2013/854623

**Published:** 2013-12-08

**Authors:** Li Chen, Yuyan Zhao, Delu Zheng, Shujing Ju, Yang Shen, Lei Guo

**Affiliations:** ^1^Department of Endocrinology, First Affiliated Hospital, China Medical University, Shenyang, Liaoning 110001, China; ^2^Department of Orthopedic Surgery, First Affiliated Hospital, China Medical University, Shenyang, Liaoning 110001, China

## Abstract

Our aim is to investigate the role of the AKT/PKB (protein kinase B) signaling pathway acting via orexin receptor 1 (OX1R) and the effects of orexin A (OXA) on cell proliferation in the insulin-secreting beta-cell line (INS-1 cells). Rat INS-1 cells were exposed to different concentrations of OXA *in vitro* and treated with OX1R antagonist (SB334867), PI3K antagonist (wortmannin), AKT antagonist (PF-04691502), or negative control. INS-1 amount of cell proliferation, viability and apoptosis, insulin secretion, OX1R protein expression, caspase-3 activity, and AKT protein levels were determined. We report that OXA (10^−10^ to 10^−6^ M) stimulates INS-1 cell proliferation and viability, reduces the proapoptotic activity of caspase-3 to protect against apoptotic cell death, and increases insulin secretion. Additionally, AKT phosphorylation was stimulated by OXA (10^−10^ to 10^−6^ M). However, the OX1R antagonist SB334867 (10^−6^ M), the PI3K antagonist wortmannin (10^−8^ M), the AKT antagonist PF-04691502 (10^−6^ M), or the combination of both abolished the effects of OXA to a certain extent. These results suggest that the upregulation of OXA-OX1R mediated by AKT activation may inhibit cell apoptosis and promote cell proliferation in INS-1 cells. This finding provides functional evidence of the biological actions of OXA in rat insulinoma cells.

## 1. Introduction

Orexin A and orexin B (OXA and OXB), also known as hypocretin-1 and hypocretin-2, are peptides that were initially discovered by orphan receptor technologies [1] and/or substrative cDNA cloning [2]. The two orexins are derived from a common prepropeptide [1, 2]. They exert biological functions by two 7-pass transmembrane receptors: orexin receptors types 1 and 2 (OX1R and OX2R) [3]. Orexins are not only restricted to the hypothalamus, but are also detected in peripheral tissues including adipose tissue, the endocrine cells of the gut, adrenal gland testis, and the pancreas [4–8]. They exert biological functions that are involved in food intake, sleep-wake behaviors, arousal, energy balance, and energy expenditure [1, 2, 9, 10]. OXA can promote pancreatic hormone secretion and reduce blood glucose levels [11, 12]. OXA and OXB have been reported with apoptosis [13, 14] and antiapoptotic [15, 16] function. OXA may act as a regulatory peptide taking part in both cell proliferation and apoptosis.

The AKT serine/threonine kinase (a.k.a protein kinase B) has been considered a critical signaling molecule within eukaryotic cells. This kinase plays an important role in a variety of physiological and pathophysiological processes in different organs systems, such as protein synthesis and transcription, angiogenesis, glycogen synthesis, and cell growth and survival [17]. Specifically, the AKT signaling pathway plays a role in regulating islet mass. Previous studies have shown that AKT-null mice have hyperglycemia and loss of *β*-cell mass with increased levels of apoptosis [18], whereas overexpression of a constitutively active AKT in *β*-cells lead to an increase in islet mass and *β*-cell proliferation [19].

In the present study, cell proliferation assays were performed to determine the effects of OXA on rat insulin-secreting *β*-cell growth. Moreover, cell death levels and caspase-3 activation were examined to analyze the effect of OXA on protection against apoptosis. Additionally, to identify the involvement of the PKB/AKT pathway, we examined the expression of total AKT and phosphorylated AKT after cells were treated with serial concentrations of OXA and inhibitors. For the first time, our data present evidence for a functional role of OXA via the OX1R-stimulated AKT signaling pathway in rat insulinoma cells.

## 2. Materials and Methods

### 2.1. Reagents

OXA was obtained from Sigma-Aldrich (St. Louis, MO, USA). RPMI Medium 1640 and bovine serum were purchased from Gibco (Grand Island, NY, USA). AKT inhibitor PF-04691502 was purchased from Cell Signaling Technology (Beverly, MA, USA). The PI3K inhibitor, wortmannin, was purchased from Sigma. The OX1R-specific antagonist SB334867 was obtained from Tocris Bioscience (Minneapolis, MA, USA). Rat insulin RIA kit was obtained from Linco Research (St Charles, Mo, USA). Total/Phospho-AKT (s473) polyclonal antibody and *β*-actin (c4) sc-47778 were obtained from Santa Cruz Biotechnology (Santa Cruz, CA, USA).

### 2.2. Cell Culture

The rat insulin-secreting beta-cell line (INS-1 cells) was obtained from American Type Culture Collection and maintained in RPMI 1640 medium supplemented with 10% (wt/vol) fetal bovine serum, l-glutamine, penicillin (50 *μ*g/mL), and streptomycin (100 *μ*g/mL). The cells were grown in a humidified atmosphere containing 5% CO_2_ at 37°C. Before the experiment, the cells were grown in petri dishes in a serum-free medium for 24 h. Next day, the cells were treated with different concentrations of OXA (10^−10^ M, 10^−8^ M, and 10^−6^ M), 10^−6^ M OXA plus 10^−6^ M PF-04691502 (AKT inhibitor), or 10^−8^ M wortmannin (PI3Ki inhibitor), 10^−6^ M SB334867 (OX1R antagonist) for 20 min, respectively.

### 2.3. Cell Viability and Proliferation

INS-1 cells were seeded (2 × 10^3^ cells/well) in 96-well plates and cultured for 24 h. To synchronize cell cycles, cells were serum-deprived for 24 h and then treated with test agents for an additional 24 h. BrdU solution (10^−6^ M) was then added and cells were incubated for 2.5 h. The BrdU Incorporation into the DNA was measured by the cell proliferation ELISA BrdU colorimetric kit (Roche Diagnostics, Penzberg, Germany); INS-1 cells were seeded into (2 × 10^3^ cells/well) well plates and cultured for 24 h. Following incubation in serum-free DMEM supplemented with various concentrations (0 M, 10^−10^ M, 10^−8^ M, and 10^−6^ M) of OXA or 10^−6^ M OXA along with 10^−6^ M OX1R antagonist SB334867 at 37°C, INS-1 cell proliferation was determined by a colorimetric methyl thiazolyl tetrazolium cell proliferation and viability assay. Fifty microliters of MTT (3-(4, 5-dimethylthiazol-2-yl)-2, 5-diphenyltetrazolium bromide, Sigma) (0.5 mg/mL MTT in PBS) cell proliferation assay solution was added to each well. After an additional 3 h, the culture medium was removed and the MTT formazan crystals were dissolved in 100 *μ*L DMSO. Optical density was measured by a plate reader (SpectraMax Plus^384^ microplate reader, Molecular Devices, Ismaning, Germany) at 570 nm and 650 nm (reference wave length). All the experiments were performed in triplicate. The A 570 nm value of control was used as a 100% standard and all individual measurements were compared to this standard.

### 2.4. Annexin V/PI Assays for Apoptosis

For Annexin V/PI assays, cells were stained with Annexin V-FITC and PI and evaluated for apoptosis by flow cytometry according to the manufacturer's protocol (BD Pharmingen, San Diego, CA, USA). Cells were treated with different concentrations of orexin A in the absence of serum for 48 h. Briefly, 1 × 10^5^ cells were washed twice with PBS and stained with 5 *μ*L of Annexin V-FITC and 10 *μ*L of PI in 500 *μ*L binding buffer for 15 min at room temperature in the dark. Quantification of apoptosis was determined by counting the number of cells stained by FITC-labeled Annexin V. Cell apoptosis was detected using the Annexin V/PI apoptosis detection kit by FACS analysis. Early apoptotic cells were identified with PI negative and FITC Annexin V positive; cells that were in late apoptosis or already dead were both FITC Annexin V and PI positive.

### 2.5. Activity of Caspase-3 in INS-1 Cells

INS-1 cells were cultured in a serum-free medium in six-well plates (1.5 × 10^5^ cells/well). Caspase-3 activity was assessed using a Caspase-3 colorimetric assay kit (BioVision Inc., Headquarters, Milpitas, CA, USA).

### 2.6. Insulin Measurements

For insulin release experiments, INS-1 cells were cultured in six-well plates until the cells were at about 80 to 85% confluence. Cells were serum-starved overnight then washed, and incubated in fresh serum-free media containing different concentrations of OXA and the different inhibitors for 24 h. At the end of the incubation period, the supernatant was taken and snap-frozen immediately in liquid nitrogen until insulin measurements were performed. The insulin levels were assessed using the ELISA kit according to the manufacturer's instructions.

### 2.7. Real-Time PCR

Total RNA was extracted from INS-1 cells using TRIzol reagent (Life Technologies Co., Carlsbad, CA, USA). The expression of OX1R and OX2R mRNA was detected by real-time PCR using TaqMan reagents (Takara, Otsu, Japan). The following specific primers were used: OX1R forward (5′-TGC GGC CAA CCC TAT CAT CTA-3′) and OX1R reverse (5′-ACC GGC TCT GCA AGG ACA A-3′); OX2R forward (5′-ATC GCA GGG TAT ATC ATC GTG TTC-3′) and OX2R-reverse (5′-TGA CTG TCC TCA TGT GGT GGT TC-3′). As an internal control for reverse transcription (RT) and reaction efficiency, amplification of glyceraldehyde-3-phosphate dehydrogenase (GAPDH) mRNA was carried out in parallel for each sample. The following specific primers were used: GAPDH forward (5′-GGC ACA GTC AAG GCT GAG AAT G-3′) and GAPDH reverse (5′-ATG GTG GTG AAG ACG CCA GTA-3′). The PCR reactions were carried out using the following conditions: 95°C for 30 s, then 40 cycles of 95°C for 5 s, 60°C for 30 s, and 95°C for 15 s. All primers and TaqMan probes specific to OX1R, OX2R, and GAPDH were designed using Primer Premier 5.0 software (Premier Biosoft International, Palo Alto, CA, USA).

### 2.8. Protein Preparations and Western Blot Analysis

INS-1 cells were washed extensively again with PBS and processed as previously mentioned to obtain total protein lysates. Cell lysates were collected and centrifuged at 12000 g for 10 min at 4°C. The supernatants were collected and mixed with 5x loading buffer and then denatured by boiling for 10 min. Samples were separated by SDS-PAGE and transferred to PVDF membranes at 60 V for 2.5 h in a transfer buffer containing 20 mM Tris, 150 mM glycine, and 20% methanol. The membranes were blocked in nonfat dry milk for 120 min at room temperature and then washed 3 times with TBST for 30 min. The PVDF membranes were incubated with primary antibody for OX1R (1 : 250) or phospho/total-TKA at a 1 : 1000 dilution in TBST overnight at 4°C. The membranes were washed and incubated with a secondary antibody for 1.5 h at room temperature, then washed three times with TBST for 30 min. The proteins were visualized by ECL. The densities were measured using Quantity-One software.

### 2.9. Statistic Analysis

The results were expressed as mean ± SEM and differences between the means were analyzed by one-way analysis of variance (ANOVA). *P* < 0.05 was considered to be statistically significant.

## 3. Results

### 3.1. Detection of OX1R Expression in INS-1 Cells

**Figure 1:**
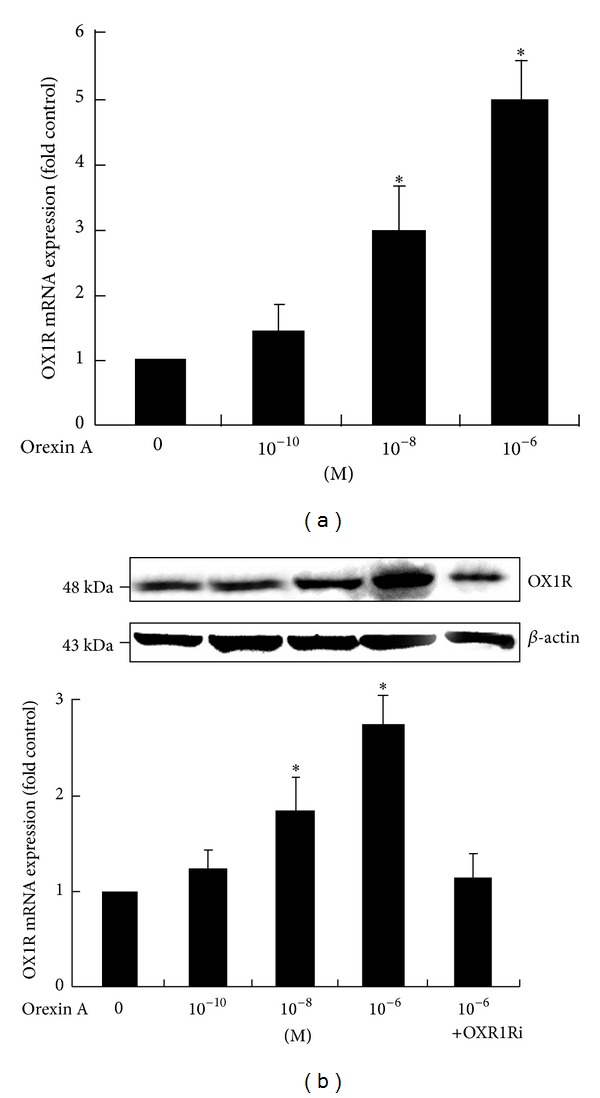
Effects of OXA on OX1R mRNA and protein expression in INS-1 cells. Cells were exposed to OXA at concentrations of 0 M, 10^−8^ M, 10^−10^ M, and 10^−6^ M for 24 h. Another treatment group consisted of 10^−6^ M OXA in the presence of the OX1R antagonist SB334867 (OX1Ri) (10^−6^ M). The expressions of OX1R mRNA (a) and protein (b) were measured via real-time PCR and western blot analysis. Data are presented as mean ± SEM based on triplicate determinations from a representative experiment. Asterisks indicate significant differences compared to control (*P < 0.05).

Real-time PCR assays demonstrated that OX1R mRNA was endogenously expressed in INS-1 cells (Figure 1(a)). However, OX2R mRNA was not detectable under the same conditions (data not shown). OXA (10^−10^ M, 10^−8^ M, and 10^−6^ M) induced a significant increase of OX1R mRNA and protein levels in a dose-dependent manner (Figures 1(a) and 1(b)). Stimulation by 10^−6^ M OXA increased OX1R mRNA and protein 5.0-fold and 2.6-fold over basal levels, respectively (*P* < 0.05). However, OXA treatment failed to stimulate OX1R protein expression in the presence of 10^−6^ M SB334867, a high-affinity OX1R-specific antagonist (Figure 1(b)).

### 3.2. Effects of OXA on Proliferation and Viability of INS-1 Cells

**Figure 2:**
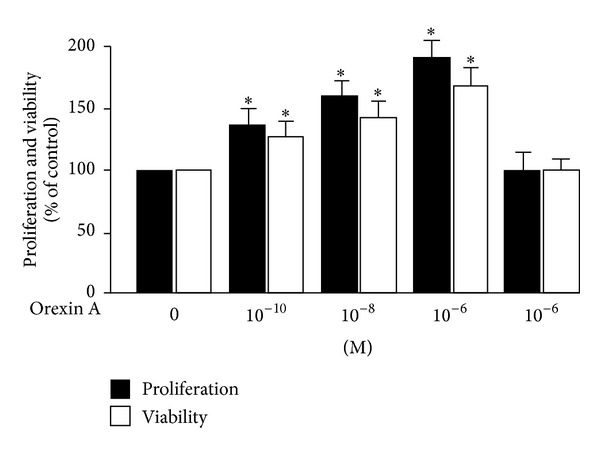
Proliferation and viability of INS-1 cells treated with OXA. Cells were treated with OXA at concentrations of 0 M, 10^−8^ M, 10^−10^ M, and 10^−6^ M for 24 h. In addition, a separate group of cells was treated with 10^−6^ M OXA in the presence of the OX1R antagonist SB334867 (OX1Ri) (10^−6^ M) for 24 h. Proliferation and viability were determined by BrdU assays and the MTT test. Data are presented as mean ± SEM based on triplicate determinations from a representative experiment. Asterisks indicate significant differences compared to control (*P < 0.05).

To determine the effects of OXA on cell viability and proliferation, INS-1 cells were stimulated with various concentrations of OXA (0 M, 10^−10^ M, 10^−8^ M, and 10^−6^ M) or 10^−6^ M OXA along with 10^−6^ M OX1R antagonist SB334867. The promoting effect of OXA on cell proliferation occurred in a concentration-dependent manner (Figure 2). Concentrations of 10^−10^, 10^−8^, and 10^−6^ M of OXA led to a 0.4-fold, 0.6-fold, and 0.8-fold increase, respectively, in cell proliferation. In cell viability, 10^−8^ M OXA and 10^−6^ M OXA caused a significant increase compared to the control. This effect was blocked by SB334867 (10^−6^ M) (Figure 2).

### 3.3. OXA Protects INS-1 Cells from Apoptosis

**Figure 3:**
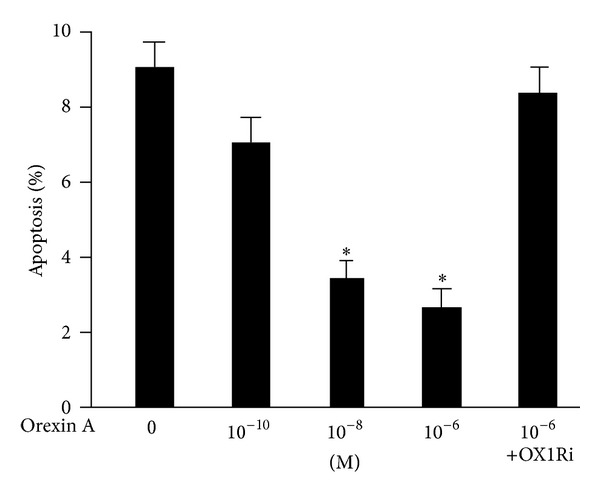
OXA protects INS-1 cells from apoptosis. Cells were exposed to OXA at concentrations of 0 M, 10^−10^ M, 10^−8^ M, and 10^−6^ M for 24 h, or cells treated with 10^−6^ M OXA in the presence of the OX1R antagonist SB334867 (OX1Ri) (10^−6^ M). Apoptosis was assessed with flow cytometry using Annexin V-FITC and PI. Data are presented as mean ± SEM based on triplicate determinations from a representative experiment. Asterisks indicate significant differences compared to control (*P < 0.05).

OXA treatment (10^−10^ M, 10^−8^ M, and 10^−6^ M) resulted in a decrease of the apoptotic index as measured by Annexin V/PI assays. Concentrations of 10^−10^ M, 10^−8^ M, and 10^−6^ M OXA led to a significant decrease in the rate of apoptosis in INS-1 cells compared to the control (*P* < 0.05) (Figure 3), but it failed to protect cells against apoptosis in the presence of 10^−6^ M SB334867 (Figure 3).

### 3.4. Effects of OXA on AKT Activation in INS-1 Cells

**Figure 4:**
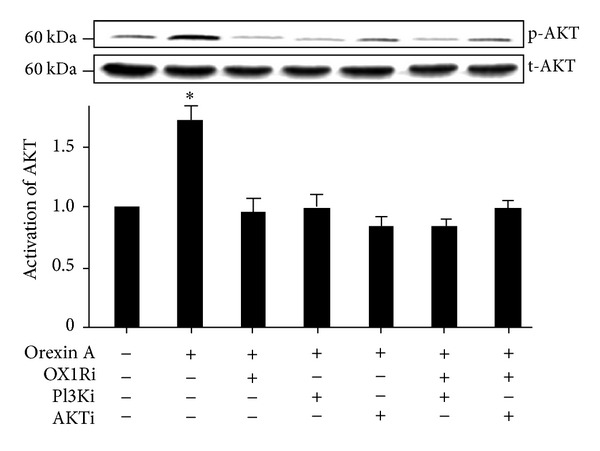
OXA increases INS-1 cell proliferation via the AKT signaling pathway. INS-1 cells were treated with OXA (10^−6^ M) for 20 min in the presence of PF-04691502 (AKTi) (10^−6^ M), wortmannin (PI3Ki) (10^−8^ M), SB334867 (OX1Ri) (10^−6^ M), or the combination of these antagonists. The phosphorylation of AKT (*p*-AKT) (corresponds to 60 kDa) was normalized against the total protein (*t*-AKT) activation. The *t*-AKT protein expression was used as an internal control for equal protein loading. Protein activation was measured by western blot analysis. Data are presented as mean ± SEM based on triplicate determinations from a representative experiment. Asterisks indicate significant differences compared to control samples (*P < 0.05).

Because the PI3K/AKT signaling pathway is involved in cell survival and apoptotic signaling, we tested whether OXA stimulation of INS-1 cells induced the activation of AKT. The data confirmed a specific increase in the *p*-AKT protein in INS-1 cells treated with 10^−6^ M OXA, compared to untreated controls (*P* < 0.05) (Figure 4), but the total AKT levels remained unaffected by OXA treatment. Moreover, the 10^−6^ M AKT antagonist PF-04691502, 10^−8^ M PI3K antagonist, wortmannin, or 10^−6^ M OX1R antagonist SB334867, as well as the combination of these antagonists, abolished the relative increase in AKT activation in response to OXA (Figure 4).

### 3.5. Effects of OXA on Proliferation and Viability of INS-1 Cells via AKT Signaling Pathway

**Figure 5:**
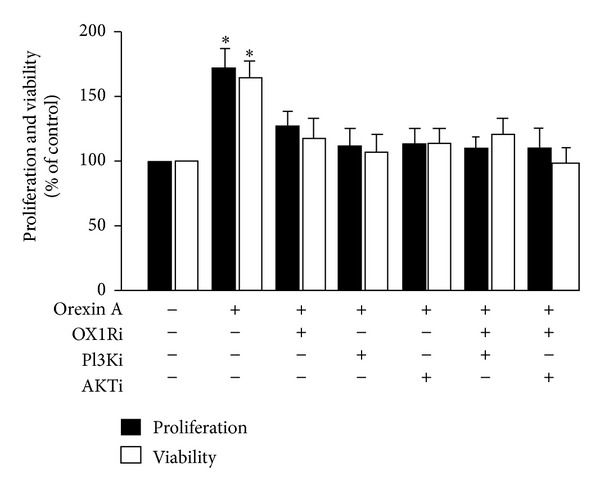
Effects of OXA on the proliferation and viability of INS-1 cells via stimulation of the AKT signaling pathway. Cells were exposed to OXA at concentrations of 0 M and 10^−8^ M for 24 h in the presence or absence of PF-04691502 (AKTi) (10^−6^ M), wortmannin (PI3Ki) (10^−8^ M), SB334867 (OX1Ri) (10^−6^ M), or a combination of these antagonists. In addition, cells were incubated with AKTi, PI3Ki, OX1Ri without OXA treatment for 24 h. Proliferation and viability were determined by BrdU assay and MTT test. Data are presented as mean ± SEM based on triplicate determinations from a representative experiment. Asterisks indicate significant differences when compared to controls (*P < 0.05).

To confirm the involvement of the AKT signaling pathway in OXA-mediated proliferation and viability in INS-1 cells, we employed BrdU analysis and MTT analysis to test cell survival. 10^−6^ M OXA significantly promoted the proliferation and viability of INS-1 cells (*P* < 0.05) (Figure 5). However, these effects were attenuated with AKT antagonist (PF-04691502) co treatment, PI3K antagonist (wortmannin), OX1R antagonist (SB334867), or the combination of these antagonists (Figure 5). Moreover, the cells were unaltered when treated with only the antagonists in the absence of OXA stimulation (Figure 5).

### 3.6. Activity of Caspase-3 in INS-1 Cells

**Figure 6:**
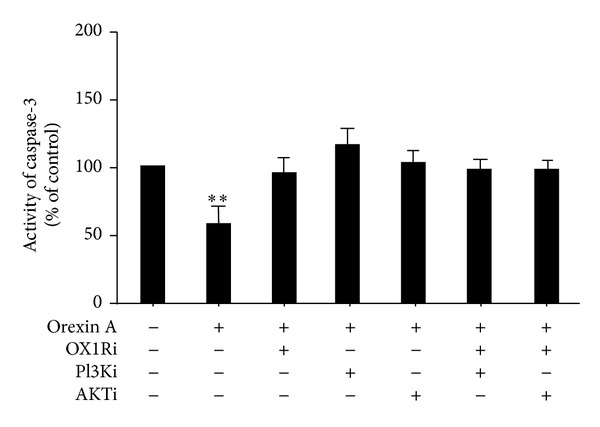
Effects of OXA on caspase-3 activity in INS-1 cells. Cells were treated with or without OXA (10^−6^ M) for 24 h in the presence or absence of PF-04691502 (AKTi) (10^−6^ M), wortmannin (PI3Ki) (10^−8^ M), SB334867 (OX1Ri) (10^−6^ M), or a combination of these antagonists. Caspase-3 activity was assessed using a caspase-3 colorimetric assay kit. Data are presented as mean ± SEM based on triplicate determinations from a representative experiment. Asterisks indicate significant differences as compared to control (**P < 0.01).

To determine whether the activation of the caspase pathway was affected in OXA-induced islet beta-cell death protection, caspase-3 activity was measured. The 10^−6^ M OXA treatment caused a significant decrease in caspase-3 activity (0.4-fold below the control) (Figure 6). This effect was blunted, however, in the presence of PF-04691502 (10^−6^ M), wortmannin (10^−8^ M) and SB334867 (10^−6^ M), as well as via the simultaneous administration of these inhibitors (Figure 6).

### 3.7. Effects of OXA on Insulin Secretion in INS-1 Cells

**Figure 7:**
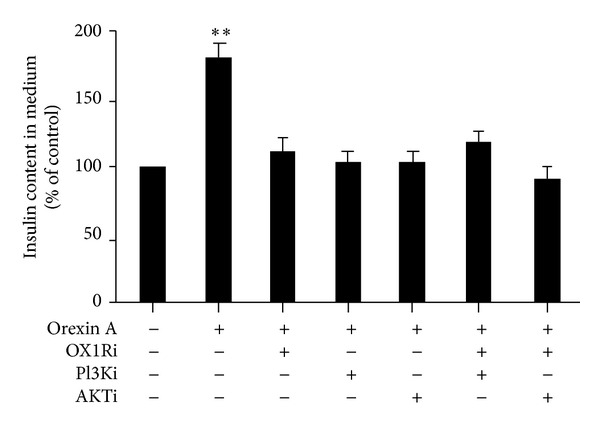
Effects of OXA on insulin secretion in INS-1 cells. Cells were exposed to 10^−6^ M OXA in the presence or absence of PF-04691502 (AKTi) (10^−6^ M), wortmannin (PI3Ki) (10^−8^ M), or SB334867 (OX1Ri) (10^−6^ M), or a combination of these antagonists. Insulin content was assessed via ELISA. Data are presented as mean ± SEM based on triplicate determinations from a representative experiment. Asterisks indicate significant differences compared to control (**P < 0.01).

After starving overnight in serum-free media, INS-1 cells were incubated with 10^−6^ M OXA and OX1R antagonist SB334867 (10^−6^ M). Treatment with 10^−6^ M OXA caused a significant increase in insulin secretion (0.8-fold compared to the control; *P* < 0.01). This effect disappeared in the presence of SB334867 (10^−6^ M) (Figure 7). Furthermore, the AKT antagonist (PF-04691502, 10^−6^ M), PI3K antagonist (wortmannin, 10^−8^ M), or OX1R antagonist (SB334867, 10^−6^ M), as well as the combination of these antagonists, abolished the relative increase in the insulin secretion in response to OXA (Figure 7).

## 4. Discussion

This study investigated the OXA-induced improvement of cell proliferation in INS-1 rat insulinoma cells. We show that OXA regulates cell viability, insulin secretion, and the activity of proapoptotic proteins in rat INS-1 cells, with this regulation likely occurring via the AKT signaling pathway.

The AKT signaling pathway is thought to mediate apoptosis, differentiation, intermediary metabolism, and proliferation, at both the transcriptional and posttranscriptional levels [20]. Some studies have shown that insulin-stimulated islet beta-cell replication is mediated by the PI3k/AKT pathway and the Erk pathway [21]. In our present study, we confirmed that OX1R is expressed in *β* islet cells, which is similar to the findings of several studies that demonstrated the expression of OX1R in the pancreas of humans and rodents [6, 22]. However, it was not clear whether OXA induced AKT activation and affected cell proliferation in pancreatic *β* cells. We observed that upregulation of AKT phosphorylation is associated with OXA stimulation, and it increases linearly with the concentration of OXA. Furthermore, the protective effects of OXA involved AKT signaling. OXA-induced cell proliferation was significantly reduced by pretreatment with different pathway antagonists such as PF-04691502, wortmannin, and SB334867, indicating that the growth and proliferation in INS-1 rat insulinoma cells likely act via the AKT signaling pathway. Interestingly, OXA treatment failed to inhibit caspase-3 activity in the presence of AKT and PI3K inhibitor, suggesting that both may be relevant in INS-1 cell proliferation process. Caspase-3 is the major effector caspase involved in a number of apoptotic pathways. Some studies show that a myriad of antiapoptotic and prosurvival substrates exist downstream of AKT [23]. The caspase-3 knockout mice were protected from caspase-3-mediated islet *β* cell apoptosis [24]. OXA may indirectly affect caspase-3 activity via OX1R through the AKT signaling pathway.

Similar to our results, previous reports have demonstrated that orexins are survival factors for various cell types. It has been shown that orexins can promote survival of neuronal cells via attenuation of caspase-3 activity [17]. In 3T3-L1 cell and 3T3-L1 preadipocytes, orexin A applied at concentrations of 10^−8^ M increased cell proliferation and survival in a concentration-dependent manner and through the ERK1/2 pathway [18, 25]. Orexins modulate the growth of cultured rat adrenocortical cells, acting via OX1R and OX2R coupled to the MAPK p42/p44- and p38-dependent cascades [26]. Thus, orexins have multifunctional activities and exert their prosurvival action by different mechanisms depending on the level of apoptotic protection. It is interesting to note that orexins are also involved in the apoptosis of various cell types including colon cancer, neuroblastoma cells, and rat pancreatic acinar AR42J cells [15, 16]. OXA-induced apoptosis is mediated, at least partly, by tyrosine phosphorylation of the immunoreceptor tyrosine-based switch motif (ITSM) and subsequent recruitment and activation of the protein tyrosine phosphatase SHP-2 [27]. Therefore, it can be hypothesized that cell neoplastic transformation might be linked with the induction of OX1R and/or OX2R expression following the activation of orexin receptor-mediated apoptosis. Nevertheless, under physiological conditions, orexins could play a part in enhanced cell proliferation and survival [28]. In this study, OXA (10^−10^ M to 10^−6^ M) is able to improve cell viability and promote insulin secretion via OX1R in INS-1 rat insulinoma cells. Our findings coincide with previous studies showing that OXA and OXB can stimulate insulin secretion from pancreatic islets via both receptor subtypes [11–14]. However, further studies are needed to clarify the mechanism by which orexin A modulates the activation of PI3K/AKT and other signaling pathways, which are crucial for INS-1 rat insulinoma cell survival and function.

In summary, our finding shows that OXA upregulates rat INS-1 insulinoma cell proliferation and reduces proapoptotic caspase-3 activity, resulting in the protection against apoptotic death via OX1R acting through the AKT signaling pathway. Because of its strong pro-survival and anti-apoptotic actions, OXA treatment may be useful for preserving islet *β*-cell function and survival.
